# A Deep Neural Network-Based Feature Fusion for Bearing Fault Diagnosis

**DOI:** 10.3390/s21010244

**Published:** 2021-01-01

**Authors:** Duy Tang Hoang, Xuan Toa Tran, Mien Van, Hee Jun Kang

**Affiliations:** 1 Department of Electrical Engineering, University of Ulsan, Ulsan 44610, Korea; hoang.duy.tang@gmail.com; 2 NTT Hi-Tech Institute, Nguyen Tat Thanh University, 300A Nguyen Tat Thanh Street, Ho Chi Minh City 70000, Vietnam; xuantoa@gmail.com; 3 Centre for Intelligent and Autonomous Manufacturing Systems, and School of Electronics, Electrical Engineering and Computer Science, Queen’s University Belfast, Belfast BT7 1NN, UK; m.van@qub.ac.uk; 4 School of Electrical Engineering, University of Ulsan, Ulsan 44610, Korea

**Keywords:** bearing fault diagnosis, deep learning, deep neural network, sensor fusion

## Abstract

This paper presents a novel method for fusing information from multiple sensor systems for bearing fault diagnosis. In the proposed method, a convolutional neural network is exploited to handle multiple signal sources simultaneously. The most important finding of this paper is that a deep neural network with wide structure can extract automatically and efficiently discriminant features from multiple sensor signals simultaneously. The feature fusion process is integrated into the deep neural network as a layer of that network. Compared to single sensor cases and other fusion techniques, the proposed method achieves superior performance in experiments with actual bearing data.

## 1. Introduction

Rolling element bearings are the most important components in rotary machines. The health condition of the bearing profoundly affects the performance, stability, and life span of the machines. Normally, when bearings have incipient defects, the machine can still operate as normal for some time. However, when the bearing defects become more serious, the breakdown of the machine or the production line is inevitable. Therefore, detecting bearing defects at the early stages is an important task in industry [1]. The integration of a real-time bearing fault diagnosis system in rotary machines helps to detect faults and predict the consequent effects in the system. From that, a plan of operating, maintaining, and repairing can be scheduled.

Presently, among existing methods, the intelligent signal-based fault diagnosis is considered to be the most popular approach. In this approach, the task of signal-based fault diagnosis is treated as a pattern classification problem, which consists of four main steps: signal acquisition, feature extraction, feature selection, and feature classification [2]. In the first step, signals are measured by different types of sensors attached to the machine. The signals can be vibration signals [3], current signals [4], acoustic emission signals [5], and so on. Since noise is inevitable in signal acquisition, the measured signals are always contaminated by noise components. Therefore, feature extraction is a critical requirement to extract only helpful information which reflects the health condition of the diagnosis object and to avoid the noise components. The feature extraction uses signal processing techniques under different domain representations of the fault signals: time domain, frequency domain, and time-frequency domain. In the time domain, statistical features are computed such as root mean square (RMS), square root of the amplitude (SRA), kurtosis value (KV), skewness value (SK), peak-to-peak value (PPV), crest factor (CF), impulse factor (IF), margin factor (MF), and so on [6]. To analyze signals in the frequency domain, Fourier transform is the most popular method [7]. In the time-frequency domain, wavelet analysis is the most popular technique which is applied extensively [8]. The output of the feature extraction step is a feature set that consists of multiple features that reflect the fault occurring in the bearing. The extracted feature set from fault signals often has a high dimension. A high number of features may reduce the performance of the successive feature classification in two factors: classification accuracy and computation time. Optionally, the feature selection can be employed to select only the most discriminant features from the original feature set. Principal component analysis (PCA) [9], independent component analysis (ICA) [10], sequential selection [6], and Fisher discriminant analysis (FDA) [11] are some representative techniques applied in feature selection. The final feature set is fed into a classifier to predict the condition of the input signal. In general, neural network (ANN) [12], support vector machine (SVM) [13], and k-nearest neighbor (kNN) [14] are the dominant techniques in contemporary work.

The traditional intelligent approach of signal-based fault diagnosis has been extensively applied to industrial applications and achieves great results. However, in this approach, some weaknesses still exist. The diagnosing performance highly depends on the feature extraction step which requires signal processing techniques, human labor, and expert knowledge. The requirement of human labor and expert knowledge means the feature extraction cannot become an automatic step. For each specific fault diagnosis task, a new feature extraction procedure must be redesigned manually. Moreover, the traditional machine learning algorithm fails in extracting highly complex feature abstraction from signals.

Deep learning (DL) or deep neural network (DNN) is a branch of machine learning (ML) which has been developed in recent years. DL exploits NNs with many layers of data-processing units. DNN can extract highly complex abstraction from data [15]. Numerous numbers of DNN models have been introduced and achieved great success in a vast number of applications. Basically, all DNN models can be considered to be the variants of four basis NNs: autoencoder [16], restricted Boltzmann machine [17], recurrent neural network [18], and convolutional neural network (CNN) [19]. Among those NNs, CNN-like models are the most popular in intelligent signal-based fault diagnosis. With the ability of effectively extracting features from signals while not requiring expert knowledge, DNNs can overcome the drawbacks of the traditional approach in signal-based fault diagnosis.

Generally, in machine fault diagnosis tasks, signals from one single sensor can produce satisfactory performance. In some cases, signals from multiple sensors can be exploited simultaneously to enhance the performance of the diagnosis systems since the multiple sensor systems may contain complementary information that is useful for the diagnosis process. Usually, it is difficult to use signals from multiple sensors to identify the status of the machines. The reason is that the signals measured by multiple sensors are disordered and correlated with multiple sources [20]. Those methods that are proposed with an attempt to use multiple data sources are called data fusion techniques. Upon the position where the fusion operation is conducted, there are three general approaches: signal-level fusion, feature-level fusion, and decision-level fusion. In machine fault diagnosis, feature-level and decision-level fusion approaches are more popular than the signal-level fusion. In the feature-level approach, principal component analysis (PCA) is often employed [21]. In general, the feature sets are combined and analyzed by the PCA algorithm to extract a new one which often has a smaller dimension. In the decision-level fusion, signals from each sensor are analyzed and classified independently and then the final decision is generated using fuzzy integral theory [22] or Dempster–Shafer (DS) theory [23,24].

It can be observed that the common point in the existing fusion techniques is the requirement of additional data-processing steps. For example, in those methods with PCA, it is necessary that a new feature set is extracted from the original data sets. Then these feature sets are combined by PCA to generate a new feature set. This final feature set is the input fed into the successive feature classification step. In those methods, which are based on DS evidence theory, first, the number of feature extractors, feature selectors, and feature classifiers are required to be equal to the number of signal sources. Moreover, it is necessary that a new decision maker is introduced to combine the results of preceding classifiers. In this paper, with the hypothesis that DNNs can effectively extract fault features from multiple signal sources, use the complementary information and eliminate the redundant one, a DNN-based fusion technique is proposed for bearing fault diagnosis. The feature-learning process is conducted in three steps. First, the input signals are represented in the time-frequency domain by using continuous wavelet transform. Second, each type of signal is extracted features by a corresponding layer of a DNN. Finally, all extracted features are fused by a layer integrated into the structure of the DNN. The novelty of this method is reflected in two points. First, the structure of the DNN is widened to simultaneously learn features from multiple sources. Second, the feature-fusing operation is integrated into the structure of the network without additional data-processing step. The effectiveness of the proposed method is verified via experiments with actual bearing data supplied by Case Western Reverse University Bearing Data Center [25].

The remainder of the paper is organized as follows. Section 2 briefly reviews sensor fusion and CNN. Section 3 explains the proposed fault diagnosis method. Experimental results and discussions are in Section 4. Section 5 concludes the paper.

## 2. Related Works

### 2.1. Sensor Fusion

Sensor fusion is the technique of unifying multiple data sources from multiple sensors to produce more consistent, accurate, and useful information than that provided by any individual data source [26]. Generally, in the topic of signal-based fault diagnosis, there are three fusion types, depending on the position on the diagnosis system where the fusion is carried out. The three types of fusion levels are as follows:

1. Signal-level fusion: this type of fusion is considered to be the lowest level where the raw signals from all sensors are combined. Since this type combines raw signals, it is necessary that all signals are comparable in a sense of data amount, sampling rate, registration, and time synchronization [22].
2. Feature-level fusion: conduct the fusion at the feature space, i.e., from each signal source (sensor), the corresponding feature set is extracted, then all feature sets are combined to generate a new one.
3. Decision-level fusion: this type is considered to be the highest level where all decisions are combined to generate a final conclusion. Based on each signal source (sensor), the health condition of the machine is predicted. Then all predictions are combined to generate the conclusion about the machine health status.

### 2.2. Convolutional Neural Network

In general, CNNs are neural networks that exploit convolutional layers which are based on convolution operation. The convolution computation between a 2-D input feature $x\in R^{m\times n}$ and a 2-D kernel $k\in R^{a\times b}$ is $y\in R^{\left(m+a-1)\times (n+b-1\right)}$:

(1) $$y\left[i,j\right]=\sum_{u=0}^{m-1}\sum_{v=0}^{n-1}k\left[u,v\right]\times x\left[i-u,j-v\right]$$

where 0⩽i⩽m+a+1 and 0⩽j⩽n+b−1. In convolutional layers, for the simplicity of the computation, kernels are often designed as square matrices, and the input features are also square matrices. Consider a convolutional layer with M kernels denoted by $k^{t}\in R^{m\times m},\;t=1:M$. Assume that this convolutional layer has the input consists of N features denoted by $x^{l}\in R^{a\times a},\;l=1:N$. Then the output of that layer will be M features, computed by:

(2) $$y^{t}=f\left(\sum_{l=1}^{l=N}k^{t}*x^{l}\right)$$

where f is the activation function. Normally, the Rectified Linear Unit (ReLU) is extensively used as an activation function because of its simplicity and less computation requirement. The math equation of ReLU is as follows:

(3) f(z)=max(z,0)

**Figure 1:**
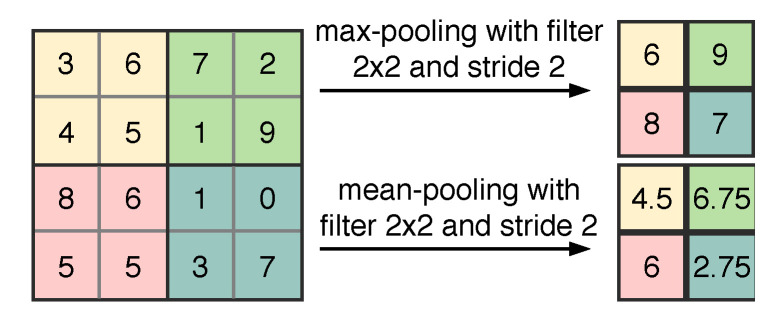
Pooling operation.

The convolutional layer is the most important layer which is the basis of the structures of a typical CNN-like neural network. Normally, after each convolutional layer, one pooling layer is implemented to reduce the spatial size of the features. In addition, this layer helps the network become invariant with a small translation of the input features [27]. The pooling operation is illustrated in Figure 1. Consider an input feature with the size of 4×4. The pooling layer can exploit the **max** or **mean** operation. The pooling layer computes the **max** (or **mean**) value of separated regions of the input matrix.

The pooling layers with the filter size of 2×2 and stride of 2 are used extensively in CNN-like NNs. With that configuration, a pooling layer with input $x^{l}\in R^{2a\times 2a}$ will have the output $y^{l}\in R^{a\times a},\;l=1:N$.

Training DNNs is often difficult because of their complex structure with many layers and a huge number of trainable parameters (weights and biases). Recently, the batch normalization (BN) technique proposed by Ioffe et al. [28] is extensively applied to accelerate the training process of DNNs. BN can improve the speed, performance, and stability of neural networks. In CNN models, BN can be implemented as a layer right after each convolutional layer. Basically, the BN layer normalizes its input features by adjusting and scaling those features. Consider N input features $x^{l}\in R^{a\times a},\;l=1:N$, BN layer computed the output features as follows:

(4) $$y^{l}=\gamma^{l}\left[\frac{x^{l}-E\left[x^{l}\right]}{\sqrt{\mathrm{Var}[x^{l}]}}\right]+\beta^{l}$$

where γ and β are trainable parameters introduced to scale and shift the normalized input features. E[x] and Var[x] denote the mean value and standard variation value of *x*, respectively. The output 2-D form features of the final pooling layer is flattened into 1-D form and fed into the dense layer. A dense layer (or fully connected layer) has the same structure with the conventional a multilayer perception, i.e., each neuron in one layer is linked to all neurons of the next layer. Flattening the input features simply rearranges the values of the input features (2-D form) into a column matrix. Consider a feature map $x^{l}\in R^{a\times a},\;l=1:N$, the output of the flattening operation will be $y\in R^{N*a^{2}\times 1}$.

The final feature map computed by the dense layer is fed into the SoftMax layer to conduct the classification. Consider a classification task with *C* labels and input feature map *x*, the SoftMax layer computes the probability of each label as follows:

(5) $$p_{j}=\frac{e^{w_{j}*x}}{\sum_{j=1}^{C}e^{w_{i}*x}}$$

The loss function of the network is computed by the cross-entropy loss as follows:

(6) $$L\left(q,p\right)=-\sum_{j=1}^{C}q_{j}\log\left(p_{j}\right)$$

where *q* is the true label of the input data.

## 3. Proposed Bearing Fault Diagnosis Method

**Figure 2:**
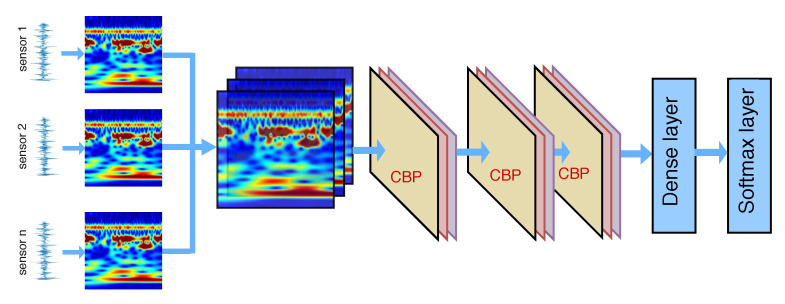
The proposed DNN.

The overall procedure of the proposed fault diagnosis method is illustrated in Figure 2. This method consists of five main steps: signal measurement, signal to image conversion, image fusion, feature extraction, and feature classification.

In the first step, the signals from various sensors are measured synchronously. These sensors can be different types or the same types but installed at different positions on the machine. Each sensor supplies a data source for the fault diagnosis system.

As shown in the previous section, the strength of CNNs is in the 2-D data form processing. Therefore, in the second step, signals are transformed into 2-D form using continuous wavelet transform (CWT). The CWT is defined as follows. A mother wavelet is a function ψ(t) with zero average (i.e., $\int_{R}\psi -0$), normalized (i.e., ||ψ||=1), and centered in the neighborhood of t=0 [29]. Scaling ψ(t) by a positive quantity *s*, and translating it by u∈R, a wavelet family can be defined as:

(7) $$\psi_{u,s}\left(t\right):=\frac{1}{\sqrt{s}}\psi \left(\frac{t-u}{s}\right),\;u\in R,\;s>0$$

Given $x\left(t\right)\in L^{2}\left(R\right)$, the continuous wavelet transform of x(t) at time *u* and scale *s* (which inversely relate to frequency) is defined as:

(8) $$W\left(s,u\right):=\langle x\left(t\right),\psi_{s,u}\rangle =\frac{1}{\sqrt{s}}\int x\left(t\right)\psi^{*}\left(\frac{t-u}{s}dt\right)$$

where $\psi^{*}$ denotes the complex conjugate of ψ. CWT decomposes the input signal x(t) into a series of wavelet coefficients. The scalogram of x(t) is defined by the function:

(9) $$S\left(s\right):=\left||W\left(s,u\right)|\right|=\sqrt{\int_{-\infty }^{+\infty }{\left|W\left(s,u\right)\right|}^{2}du}$$

If a time interval $\left[t_{0},t_{1}\right]$ needs to be considered, the corresponding windowed scalogram is defined by the function:

(10) $$S_{\left[t_{0},t_{1}\right]}\left(s\right):={\left||W\left(s,u\right)|\right|}_{\left[t_{0},t_{1}\right]}=\sqrt{\int_{t_{0}}^{t_{1}}{\left|W\left(s,u\right)\right|}^{2}du}$$

**Figure 3:**
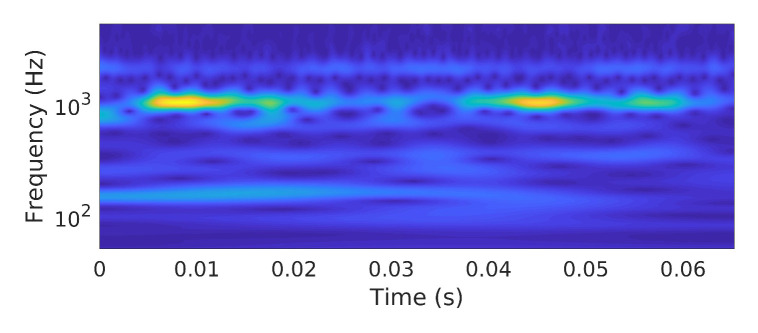
Time-frequency representation of vibration signal.

In other words, the scalogram is the absolute value of the CWT of a signal, plotted as a function of time and frequency, as shown in Figure 3.

By representing signals in the time-frequency domain as image form, the health condition of the machine will be reflected by a data sample which consists of *n* images corresponding to *n* signal sensors. The task of bearing fault diagnosis now can be considered to be the task of image classification. However, this image classification task cannot be solved by the conventional CNN since each data sample consists of multiple images. Therefore, a new model of DNN is proposed to handle the multiple-image data samples. Assume that there are *n* data sources, corresponding to *n* sensors used for the diagnosis in the rotary machine. Based on the number of data sources, the proposed DNN model has *n* branches accordingly, each branch will handle one individual image in the data sample which is fed into the network. Each branch consists of multiple successive convolutional layer–batch normalization layer–pooling layer (a CBP module). Each branch takes the role of extracting features from the corresponding image data source.

The output of each branch is considered to be a feature set of the corresponding signal source. All feature sets are combined at the feature-fusing layer, which is integrated as a layer in the network. Assume that there are *n* signal source. Accordingly, there are *n* feature sets, denoted by $X_{i},\;i=1:n$. Each feature set has a size of h×m×m. The output of the feature-fusing layer is a new feature set with a size of $1\times hnm^{2}$. The new feature set is then fed into the dense layer to learn higher level features. Finally, the output feature set from the dense layer is classified by the SoftMax layer. The final prediction is made based on the output probabilities of the SoftMax layer.

## 4. Experiments

### 4.1. Test-Bed and Data Preparation

The actual bearing fault data are supplied by the Bearing Data Center of Case Western Reverse University. The test-bed consists of a 2-hp motor, a torque transducer, and a dynamometer. The test bearings support the motor shaft. The test bearings were seeded with faults using electro-discharge machining (EMD). The bearing test-bed can be operated under different load values by changing the torque applied to the motor. Four operating conditions are considered include 0 hp, 1 hp, 2 hp, and 3 hp.

Vibration signals are measured by two accelerometers placed at the fan-end (FE) and drive-end (DE) of the motor. Two accelerators measure vibration signals simultaneously and the signals are digitized at sampling frequency 12 kHz. There are four types of bearing conditions considered, including one type of healthy bearing and three types of fault bearings: bearing with fault at inner race fault, bearing with fault at outer race fault, and bearing with fault at rolling elements. Each type of bearing fault has different fault diameter, including 7 mils (mili-inches), 14 mils, and 21 mils. Totally, as shown in Table 1, ten types of bearing conditions are labeled from 0 to 9, respectively.

Since vibration signals are measured by two accelerators FE and DE simultaneously, each bearing condition is reflected via two separate files. Overall, 20 vibration signal files are recorded corresponding to 10 bearing conditions.

### 4.2. Other Methods for Comparison

To evaluate the effectiveness of the proposed method, as well as analyze the advantages and disadvantages, some other bearing fault diagnosis methods published recently have been adopted to make comparisons. The proposed method in this paper is a combination of deep learning and sensor fusion. Therefore, it is necessary to compare it with the deep learning-based approaches and the sensor fusion approaches. The first method to compare is published in [30]. In this method, a DNN based on LeNet-5 is developed to classify a gray-scale image data-set, which is obtained by transforming the vibration signals. The second method which is taken into account is published in [31]. In this method the transfer learning technique is adopted to reuse the Alexnet, a well-known DNN in image classification [32]. Originally, Alexnet is a very deep NN trained for image classification. When adopted into the new task of bearing fault diagnosis, it is re-trained with the time-frequency image of vibration fault signals. The third method adopted to compare is a PCA-based fusion method. In this method, vibration signals are transformed into the time-frequency domain. From each sample of the signal, 14 features are extracted. Therefore, each bearing condition is indicated by a total of 28 features (14 features from the DE sensor and 14 features from the FE sensor). PCA algorithm is exploited to fuse the two feature sets and generate a new one consisting of only 20 features. Finally, MLP is employed to classify the fused feature set generated by PCA. The fourth method to compare is based on the DS evidence theory. In this method, two separated NNs are used to diagnose the bearing condition using DE and FE signals, respectively. Then the DS evidence theory is manipulated to combine the diagnosing results of the NNs. In this method, the fusion is conducted at the decision level, while in the PCA-based method, the fusions are conducted at the feature level.

### 4.3. Signal Pre-Processing

The DNN models for the fault diagnosis problem need to be trained by a significant amount of data samples. Therefore, the signal files are split into equal segments. As mentioned in Section 4.2, the method proposed in [30] is taken into account for comparison. In this method, there is a step where the signal samples are rearranged to form gray images and apply the image classification by LeNet5. Therefore, an MNIST-like data-set is going to be created. In the MNIST data-set, the gray images have a size of 28×28. Therefore, the length of signal segments is selected at value 28×28=784. For each condition of the test bearing, 600 signal segments are prepared, including 300 segments of vibration signal measured by FE sensor and 300 segments of vibration signal measured by the DE sensor.

**Figure 4:**
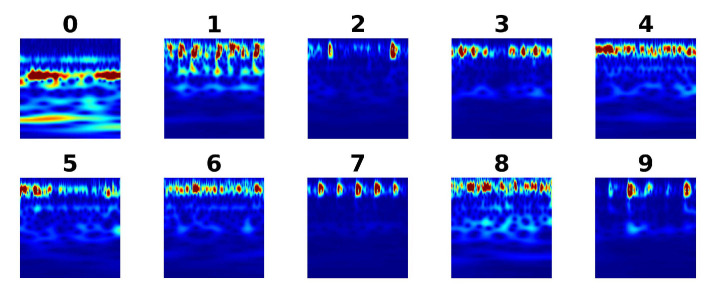
Time-frequency representations of vibration signals.

The next step in signal pre-processing is to represent signal segments in the time-frequency domain using CWT. The original time domain vibration signals are represented in the time-frequency domain using CWT, which exploits the Morse wavelet function. The time-frequency images of vibration signals are shown in Figure 4. In each condition of bearing, the training data-set and testing data-set are split randomly with the ratio of 7/3.

### 4.4. Designing and Training the Proposed DNN

In the topic of image classification, many well-known DNNs have been successful with an image size of 3×224×224. Inspired by that fact, in this paper, all time-frequency images are resized to a size of 3×224×224. As a result, the inputs of DNNs have the size of 3×224×224. The convolution kernel size and stride step length were determined by trial-and-error process, taking into the consideration of the values used in the literature. The kernel size of 3×3 with the padding zero technique and the stride step of 1 is used in all convolutional layers. In all pooling layers, the kernel size of 2×2 and the stride step of 2 are used. Consequently, after passing a group of three successive layer: convolutional layer, batch normalization layer, and pooling layer, the output data will have the number of feature maps equal to the number of the kernels in the convolutional layer, and the size of feature size is reduced by 2.

The feature-fusing layer flattens the two input feature sets of two branches, and concatenates them to generate a fused feature map. Then this feature map is fed into the dense layer, and finally, the SoftMax layer classifies the feature map to predict the class of the input data sample. It is noticeable that each data sample consists of two time-frequency representations (from DE and FE sensors). The fused feature set has a size of 1 × 12,544.

The SoftMax layer of the DNN has 10 outputs, corresponding to 10 classes of bearing health conditions that need to be classified. The network is trained with mini batch stochastic gradient descent with momentum algorithm, the learning rate α=0.001, the momentum β=0.9, and the batch size 16.

### 4.5. Fault Diagnosis Results

All methods mentioned in Section 4.2 are taken into account to make a comparison with the proposed method. Among those methods, the method using Lenet5 and Alexnet uses single signal sources. These two methods will be evaluated by both the DE signal source and the FE signal source separately. The PCA-based, DS-based, and the proposed method in this paper are fusion-based methods. Therefore, the DE and FE signal sources will be used simultaneously for these three methods. Each method is trained by 2100 data samples and tested by 700 data samples. The accuracy of diagnosis with 700 testing data samples is the criteria to evaluate the performance of those fault diagnosis methods. Four different operating conditions of the test bearing are taken into account for evaluating the fault diagnosis methods. The accuracy of all methods is shown in Table 2. It can be observed that all methods, under all operating conditions of the test bearing achieve very high accuracy. However, the results of the DS-based fusion method are slightly lower the others.

### 4.6. Evaluation under Noisy Conditions

**Figure 5:**
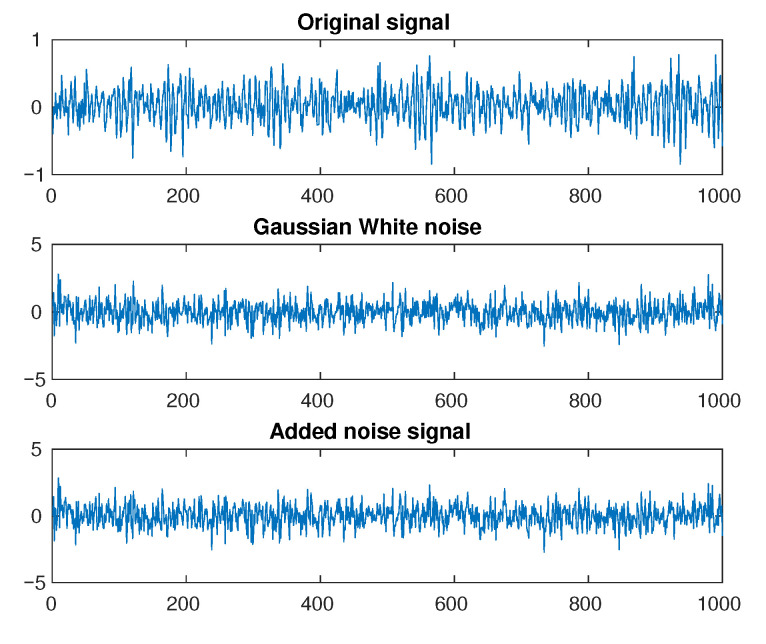
Add noise to original signal.

In industrial environments, the sensory signals are always contaminated by noise. That leads to the degradation of the performance of the fault diagnosis system. To evaluate the robustness against the noise of fault diagnosis methods, noise signals are introduced into the original vibration signals. The additive Gaussian white noise (AGWN) with various standard variances is added to the original vibration signals. The signal-to-noise ratio (SNR) is defined as follows:

(11) $$SNR=10\log\left(\frac{P_{signal}}{P_{noise}}\right)$$

where $P_{signal}$ and $P_{noise}$ are the power of signal and noise, respectively. Figure 5 shows the noisy signal made by adding an original signal with Gaussian white noise. The experiments are conducted with noisy signals which obtained by adding AWGN with different SNR values vary in the range [−1,−2,−3,−4,−5,−6,−7,−8].

**Figure 6:**
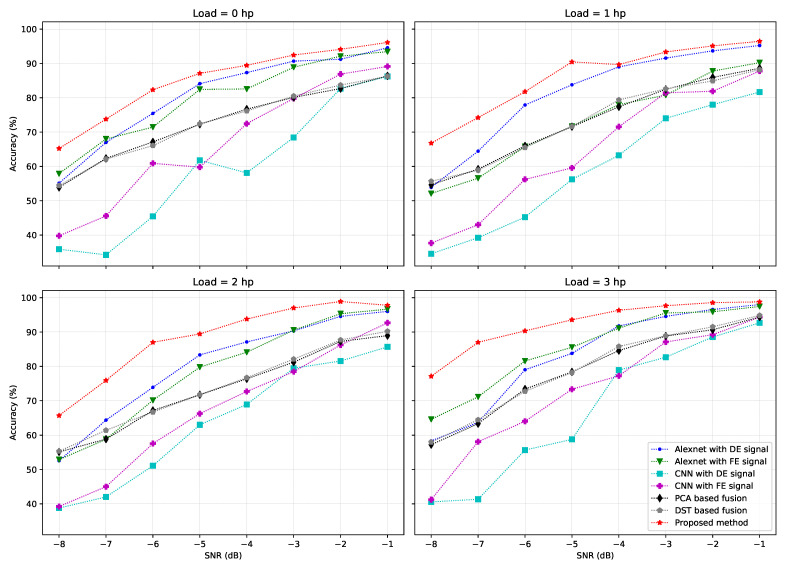
Evaluation of fault diagnosis methods under different SNRs and operating conditions.

Figure 6 and Table 3 show the experiment results. Table 3 describes the experiment results conducted under different working conditions. We consider 4 load conditions: 0 hp to 1 hp, 2 hp, and 3 hp. Under each load condition, the noise levels are changed from −8 dB to −1 dB. The classification accuracy is the metric used to compared 7 fault diagnosis methods, included the proposed one. It can be observed that the common trend of all diagnosis methods is the more noise added to the original signal, the worse the classification accuracy. In the worst case (−8 dB noise level), some methods cannot exceed 50% accuracy. Among all compared methods, the proposed method achieves the highest accuracy under all working conditions. Figure 6 shows some facts as follows:

- The diagnosis accuracy of all methods decreases in accordance with the noise level in the signal.
- For the two methods using single signal source, the diagnosis accuracy in the case of DE sensor and FE sensor are very close.
- The PCA-based and DS-based fusion methods have very similar diagnosis accuracy, since in these two methods, the same feature set is extracted.
- Among the compared methods, the proposed method which uses the DNN-based feature fusion has the most consistency against noise, better than all other methods.

## 5. Conclusions

This paper proposed a novel deep neural network for the task of multiple sensor-based fault diagnosis. The proposed DNNs with wide structure and integration of the feature fusion as a network layer help to learn features from multiple signal sources simultaneously and effectively. As a result, the proposed method can achieve better results compared to other DL-based and fusion-based methods, especially under noisy conditions. The proposed neural network is not limited in the topic of this paper, i.e., used for vibration signals, it can be applied to any fault diagnosis task that have to fuse multiple signal sources. In future works, we will evaluate the proposed method with other types of signals such as current signals and acoustic emission signals. Moreover, we will also examine the performance of the proposed method for the case of fusing different types of signals in the same fault diagnosis system.

## Figures and Tables

**Table 1:** Labeling for signal.

Bearing Conditions	Fault Size (Mils)	Label
No fault		0
Inner race fault	7	1
Inner race fault	14	2
Inner race fault	21	3
Ball fault	7	4
Ball fault	14	5
Ball fault	21	6
Outer race fault	7	7
Outer race fault	14	8
Outer race fault	21	9

**Table 2:** Fault diagnosis accuracy of methods.

Method	Sensor	Accuracy (%)			
		0 hp	1 hp	2 hp	3 hp
Alexnet	DE	100.0	99.78	100.0	100.0
Alexnet	FE	100.0	99.78	99.89	100.0
Lenet5	DE	97.56	96.67	99.56	99.67
Lenet5	FE	97.56	96.67	99.56	99.67
PCA-based fusion	DE and FE	98.6	98.6	99.25	99.4
DS-based fusion	DE and FE	97.73	97.67	99.05	99.47
Proposed method	DE and FE	100.0	99.56	100.0	99.78

**Table 3:** The diagnosis accuracy (%) of methods under different working conditions.

**Noise Level (dB)**	**−8**	**−7**	**−6**	**−5**	**−4**	**−3**	**−2**	**−1**	Load 0 hp
Alexnet-DE	55.11	67.0	75.44	84.11	87.33	90.67	91.22	94.56	
Alexnet-FE	57.89	68.0	71.44	82.44	82.56	88.89	92.11	93.44	
Lenet5-DE	35.89	34.22	45.44	61.78	58.11	68.44	82.67	86.22	
Lenet5-FE	39.78	45.56	60.89	59.78	72.44	79.78	86.89	89.11	
PCA-based fusion	53.88	62.33	67.14	72.27	76.68	80.1	82.68	86.37	
DS-based fusion	54.42	62.13	66.06	72.4	76.14	80.5	83.69	86.24	
Proposed method	65.11	78.11	82.78	85.33	90.0	92.22	94.44	96.22	
Alexnet-DE	53.89	64.44	77.89	83.78	89.0	91.56	93.67	95.22	Load 1 hp
Alexnet-FE	52.11	56.56	65.78	71.67	78.0	80.78	87.78	90.22	
Lenet5-DE	34.56	39.22	45.22	56.22	63.22	74.0	78.0	81.67	
Lenet5-FE	37.67	43.0	56.22	59.56	71.56	81.44	81.89	87.78	
PCA-based fusion	54.66	59.2	65.98	71.57	77.36	82.46	85.93	88.59	
DS-based fusion	54.66	59.2	65.98	71.57	77.36	82.46	85.93	88.59	
Proposed method	53.22	73.44	83.33	90.0	91.89	93.11	96.44	97.44	
Alexnet-DE	52.56	64.33	73.89	83.33	87.11	90.33	94.56	96.0	Load 2 hp
Alexnet-FE	52.89	58.78	70.11	79.78	84.11	90.56	95.33	96.67	
Lenet5-DE	38.78	42	51.11	63	68.89	79.22	81.56	85.67	
Lenet5-FE	39.22	45	57.57	65.67	72.67	78.44	86	92.33	
PCA-based fusion	55.12	58.85	67.14	71.73	76.36	81.21	87.27	88.94	
DS-based fusion	55.39	60.83	66.4	71.8	76.9	82.09	87.48	90.21	
Proposed method	75.11	78.67	89.78	93.11	96.33	97.56	98.78	99.11	
Alexnet-DE	58.33	63.67	79.0	83.78	91.78	94.56	96.56	98.0	Load 3 hp
Alexnet-FE	64.56	71.11	81.56	85.56	91.11	95.56	95.89	97.44	
Lenet5-DE	40.56	41.33	55.67	58.78	78.89	82.67	88.56	92.67	
Lenet5-FE	41.22	58.11	64.0	73.33	77.22	87.11	89.22	94.33	
PCA-based fusion	57.25	63.35	73.39	78.39	84.57	88.83	90.66	94.36	
DS-based fusion	57.98	64.42	72.72	78.18	85.79	88.9	91.52	94.83	
Proposed method	79.89	83.89	92.67	96.0	96.78	97.67	98.44	98.89	

## Data Availability

Data is available on Reference [25].

## Abbreviations

List of Abbreviation used in paper:

ANN	artificial neural network
CF	crest factor
CNN	convolutional neural network
CWT	continuous wavelet transform
DNN	deep neural network
DL	deep learning
FDA	fisher discriminant analysis
ICA	independent component analysis
IF	impulse factor
kNN	k-nearest neighbor
KV	kurtosis value
MF	margin factor
PCA	principal component analysis
PPV	peak-to-peak value
RMS	root mean square
SK	skewness value
SNR	signal-to-noise ratio
SRA	square root of the amplitude
SVM	support vector machine
